# The Effects of Different Degrees of Carbohydrate Restriction and Carbohydrate Replacement on Cardiometabolic Risk Markers in Humans—A Systematic Review and Meta-Analysis

**DOI:** 10.3390/nu12040991

**Published:** 2020-04-02

**Authors:** Eva Fechner, Ellen T.H.C. Smeets, Patrick Schrauwen, Ronald P. Mensink

**Affiliations:** Department of Nutrition and Movement Sciences, NUTRIM School of Nutrition and Translational Research in Metabolism, Maastricht University Medical Center, PO Box 616, 6200 MD Maastricht, The Netherlands; e.fechner@maastrichtuniversity.nl (E.F.); p.schrauwen@maastrichtuniversity.nl (P.S.)

**Keywords:** meta-analysis, adults, randomized controlled trials, low-carbohydrate diets, LCD, carbohydrate restriction, cardiovascular risk

## Abstract

Low-carbohydrate diets (LCDs) often differ in their diet composition, which may lead to conflicting results between randomized controlled trials. Therefore, we aimed to compare the effects of different degrees of carbohydrate (CHO) restriction on cardiometabolic risk markers in humans. The experimental LCDs of 37 human trials were classified as (1) moderate-low CHO diets (<45–40 E%, *n* = 13), (2) low CHO diets (<40–30 E%, *n* = 16), and (3) very-low CHO diets (<30–3 E%; *n* = 8). Summary estimates of weighted mean differences (WMDs) in selected risk markers were calculated using random-effect meta-analyses. Differences between the LCD groups were assessed with univariate meta-regression analyses. Overall, the LCDs resulted in significant weight loss, reduced diastolic blood pressure BP, and increased total cholesterol and high-density lipoprotein cholesterol (HDL-C), without significant differences between the three LCD groups. Higher low-density lipoprotein cholesterol (LDL-C) concentrations were found with the very-low CHO diets compared to the moderate-low CHO diets. Decreases in triacylglycerol (TAG) concentrations were more pronounced with the low and very-low CHO diets, compared to the moderate-low CHO diets. Substitution of CHO by mainly saturated fatty acids (SFAs) increased total cholesterol, LDL-C, and HDL-C concentrations. Except for LDL-C and TAGs, effects were not related to the degree of CHO restriction. Potential effects of nutrient exchanges should be considered when following LCDs.

## 1. Introduction

Low-carbohydrate diets (LCDs) have been proposed in the management of obesity, diabetes, and cardiovascular diseases (CVD) [1,2,3]. However, randomized controlled trials (RCTs) have shown conflicting results on the effects of LCDs on weight loss, glycemic control, and serum lipid profiles, while positive effects have mainly been observed in short-term studies [4]. The European Food Safety Authority therefore concluded that scientific evidence is not sufficient to advocate either low or high carbohydrate (CHO) diets and thus recommends a range for CHO-intake between 45% to 60% of total energy (E%), including a daily fiber intake of at least 25 grams [5]. Diets providing less than 45 E% from CHO could therefore be referred to as LCDs [6]. 

In the human intervention trials performed so far, intakes of CHO differed widely between studies, which also implies varying amounts of fats and proteins. It is well-known that different degrees of CHO restriction may result in different metabolic effects [6]. For example, while LCDs providing more than 50 g of CHO per day prevent dietary ketosis in most people, a very-low carbohydrate ketogenic diet (VLCKD) providing less than 50 grams CHO per day typically leads to the production of measurable amounts of ketone bodies in the urine [7]. The metabolic switch to ketosis may result in immediate side-effects like loss of appetite [8] or in long-term side effects, such as kidney stones [9]. Johnston et al. [10] compared a ketogenic (9 E% CHO) with a non-ketogenic LCD (42 E% CHO) under isocaloric conditions and concluded that a lower CHO content and the initiation of dietary ketosis did not provide a metabolic advantage over the non-ketogenic LCD. In contrast, Saslow et al. [11] found a higher weight loss and improved glycemic control when comparing an energy-restricted VLCKD (14 E% CHO) to an isocaloric energy-restricted LCD (41 E% CHO). More recently, Harvey et al. [12] compared three LCDs containing 5 E%, 15 E%, and 25 E% of CHO. Compared to baseline, significant decreases in body weight and triacylglycerol (TAG) concentrations were observed, while total cholesterol, low-density lipoprotein cholesterol (LDL-C), and high-density lipoprotein cholesterol (HDL-C) concentrations were increased after 12 weeks of CHO restriction. However, changes did not differ between the diet groups. Still, RCTs comparing different types of LCDs are limited and no systematic overviews, evaluating the degree of CHO restriction on metabolic risk markers, have been published yet [12].

The main objective of this systematic review and meta-analysis was therefore to compare the effects of different degrees of CHO restriction on conventional fasting cardiometabolic risk markers and to investigate whether dose-dependent relationships were present. Under isocaloric conditions, reducing CHO intake is accompanied by increases in fat and/or protein intake. In addition, the fatty-acid composition, as well as cholesterol and fiber intakes may change, which can affect glucose and lipid metabolism [13,14,15]. Therefore, not only the amount of CHO was addressed, but also the macronutrients used to substitute CHO. To make studies more comparable and to avoid the comparison to high-carbohydrate diets (HCD)/low fat diets (LFDs), only studies that compared LCDs to moderate-carbohydrate diets (MCDs, 45–55 E%) were included.

## 2. Methods

The Preferred Reporting Items for Systematic Reviews and Meta-Analyses (PRISMA) checklist and flow-diagram for reporting systematic reviews and meta-analyses [16] were used as tools to structure this review, to describe the used methodology, and to systematically present our findings.

### 2.1. Search Strategy

Potentially relevant studies were identified in April 2019 by a systematic literature search in three databases (Ovid Medline, Embase, and the Cochrane Central Register of Clinical Trials). The following search terms were used: ‘low carbohydrate diet or carbohydrate restricted diet or very low carbohydrate diet or ketogenic diet or very low carbohydrate ketogenic diet or low glycemic load diet or high fat diet or high protein diet’ (MeSH term) and ‘weight or BMI or glucose or insulin or triacylglycerol or triacylglycerol or cholesterol or HDL or LDL or blood pressure’ (MeSH term) and ‘randomized controlled trial or comparative study or controlled clinical trial’ [publication type] and ‘humans’ [Subjects].

### 2.2. Study Selection

The selection procedure was divided into two stages. An initial selection was made by screening titles and abstracts only, followed by a second full text selection of the remaining articles. Articles were included when they did meet the following criteria: (a) randomized controlled intervention trial in free-living adult human subjects; (b) comparison of the effects of a LCD (<45 E%) with those of a MCD (45–55 E%); (c) no use of nutritional supplements or meal replacers, or life style changes like exercise; (d) measurement of at least one of the following cardiometabolic risk factors: weight, fasting total cholesterol, HDL-C, LDL-C, TAGs, glucose, insulin, or blood pressure (BP); (e) reported daily nutrient intake based on food diaries or recalls; (f) not intermediate measurements; and (g) full text available in English. The study selection was performed by two authors (EF and RPM). When inconclusive, the eligibility of the studies was discussed until consensus was reached.

### 2.3. Data Collection

Data was extracted from each of the selected articles and summarized in a spreadsheet. Information regarding study design, duration, participants, diet composition, and the available data on the above-mentioned cardiometabolic risk markers was extracted. Nutrients were expressed as energy percentages (E%), except for intakes of energy, cholesterol, and fiber which were expressed in kcal, milligrams, and grams, respectively. If needed, plasma and serum markers (total cholesterol, LDL-C, HDL-C, TAG, and glucose) were converted to mmol/L and insulin concentrations to μU/mL. Based on CHO intake, studies were allocated to one of the three different groups: moderate-low CHO diets (group 1, <45–40 E% CHO), low CHO diets (group 2, <40–30 E%), and very-low CHO diets (group 3, <30–3 E%). These three groups were defined after inventory of the included studies in order to create comparable group sizes. 

### 2.4. Statistical Analyses

All statistical analyses were performed with Stata 12.1 software (Stata Corporation, College Station, TX, USA). For cross-over studies, diet effects were calculated by subtracting the mean value at the end of the control period from the mean value at the end of the intervention period. For parallel-designed studies, mean changes in the control group were subtracted from the mean changes in the intervention group. Mean changes were defined as the difference between the measurements before and after treatment. The same procedure was followed for calculating the differences in nutrient intakes. Multiple study arms were included when the amounts of CHO in treatment and control groups corresponded to the inclusion criteria. 

Summary estimates of weighted mean differences (WMDs) in weight, total cholesterol, LDL-C, HDL-C, TAGs, glucose, insulin, systolic and diastolic BP, and 95% confidence intervals were determined using fixed-effect meta-analyses and visualized using forest plots. As weighing factor, the inverse of the between-subject variance was used (1/SE^2^). For each outcome parameter, heterogeneity between studies was assessed with the Cochran’s Q test and expressed as the I^2^ statistic [17]. The I^2^ statistic is the percentage of variability in the effect estimate caused by heterogeneity. In case of relevant heterogeneity between studies (I^2^ > 50%), random-effect meta-analyses were performed. Subgroup analyses were performed to evaluate changes in body weight, saturated fatty acid (SFA) and protein intakes, the health status of the study population (healthy, overweight/obese, or metabolically impaired), study design (parallel and cross-over), and study duration (≤1 month, ≤6 months, and >6 months), as potential sources of heterogeneity between studies. For the continuous variables (changes in body weight, SFA and protein intakes), the median effect estimates were used as cut-off values to create two subgroups within each variable, e.g., lower or equal to vs. higher than the median changes.

To assess differences in the selected outcome parameters between the CHO groups, univariate meta-regression analyses were performed. The CHO groups were treated as factor variables and two-sided tests were performed, using the moderate-low CHO group as a reference. A *p*-value of <0.05 was considered as statistically significant. 

For all parameters, publication bias was determined within each CHO group by visual inspection of funnel plots and by assessing funnel plot asymmetry with the Egger’s weighted regression test [18]. Absence of publication bias is indicated by an intercept close to 0 with a corresponding *p*-value ≥ 0.05. Funnel plot asymmetry was not detected for any of the outcome parameters within the three CHO groups, as shown by insignificant results of the Egger’s test (data not shown). Therefore, it can be assumed that no publication was present based on the selected studies in this review.

## 3. Results and Discussion

**Figure 1:**
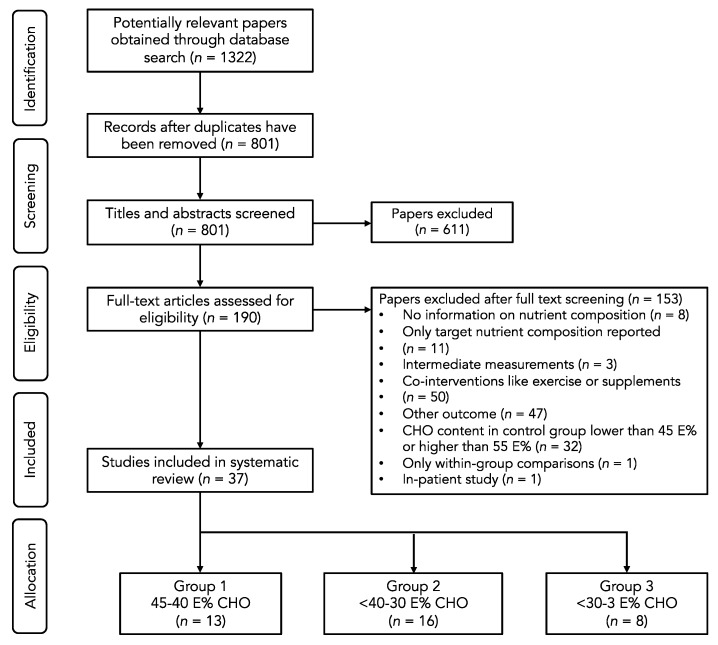
Flow diagram of the selection process.

The systematic database search retrieved 1322 potentially relevant papers. After title and abstract screening, 190 papers remained for the full text screening. Finally, 37 papers were included in the systematic review. A flowchart of the study selection process is presented in Figure 1.

### 3.1. Group 1: Effects of Moderate-Low CHO Diets (<45–40 E%)

Thirteen studies were identified that compared the effects of a moderate-low CHO (<45–40 E%) to an MCD (45–55 E%). Four studies were carried out in type 2 diabetes patients [19,20,21,22], four studies in overweight or obese participants [23,24,25,26], two studies in non-obese men with elevated TAGs [27,28], and three studies in healthy volunteers [29,30,31]. Study durations varied between 3 weeks and 18 months. Study characteristics are shown in Table 1.

**Figure 2:**
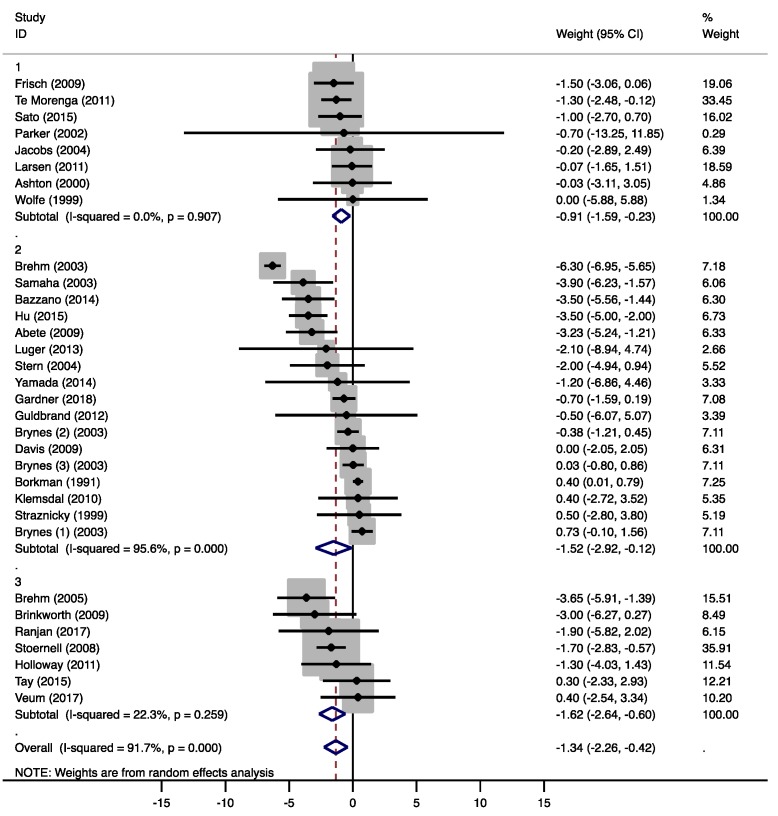
Forest plots of randomized controlled trials that examined the effects of carbohydrate (CHO) restriction on body weight changes. Studies were categorized in group 1 (moderate-low CHO, 40–45 E%), group 2 (low CHO, 40–30 E%), and group 3 (very-low CHO, 30–3 E%). Solid squares represent the weight of individual studies and diamonds represent the weighted mean difference (WMD) in weight change. Effects were calculated using random-effect meta-analysis. No significant differences in body weight changes were detected between the low-carbohydrate diet (LCD) groups.

Overall, the moderate-low CHO diets resulted in significant weight loss (−0.91 kg, 95% CI −1.59 to −0.23 kg, *p* = 0.009, Figure 2). In five studies [20,22,23,24,25], energy restriction was intended in both diet groups, while eight studies intended to keep energy intake stable on both diets [19,21,26,27,28,29,30,31]. Of the eleven studies that reported changes in body weight, only two studies showed a significant weight reduction in the moderate-low CHO group compared to the MCD group [19,24]. Six studies [20,22,23,25,26,27] did not find significant differences between both groups, but reported comparable weight losses due to a lower energy intake in both diet groups. Three studies [21,30,31] found no changes in body weight in both intervention groups. These studies kept energy intake stable during the study. 

**Figure 3:**
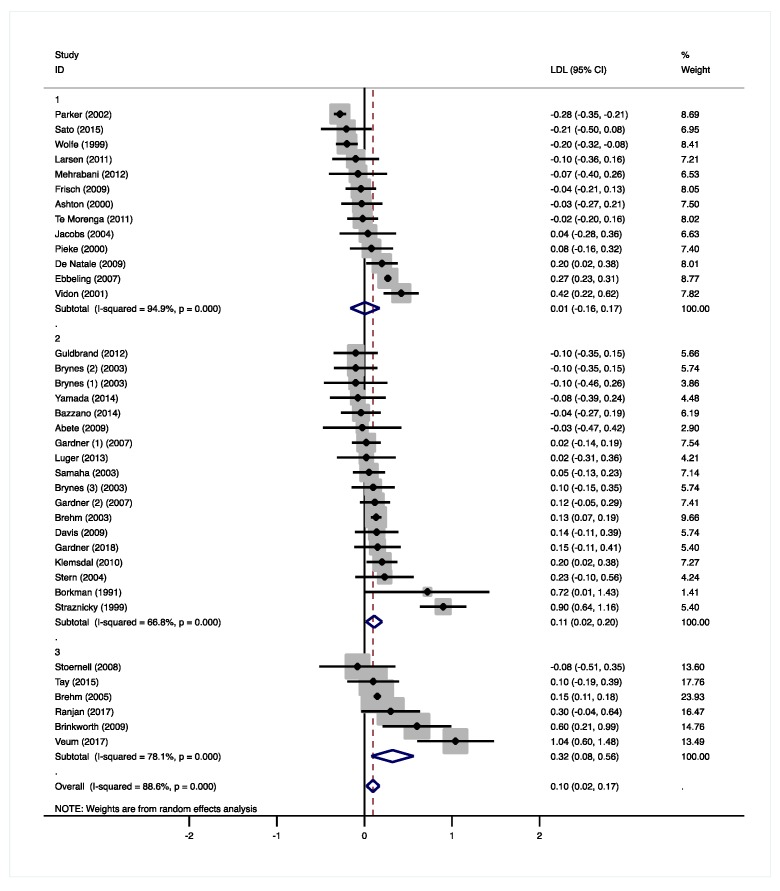
Forest plots of randomized controlled trials that examined the effects of carbohydrate (CHO) restriction on low-density lipoprotein cholesterol (LDL-C) concentrations. Studies were categorized in group 1 (moderate-low CHO, 40–45 E%), group 2 (low CHO, 40–30 E%), and group 3 (very-low CHO, 30–3 E%). Solid squares represent the weight of individual studies and diamonds represent the weighted mean difference (WMD) in LDL-C. Effects were calculated using random-effect meta-analysis. Decreases in LDL-C were significantly larger in the very-low CHO compared to the moderate-low CHO group.

The moderate-low CHO diets did not result in significant changes in total cholesterol (−0.05 mmol/L, 95% CI −0.19 to 0.08 mmol/L, *p* = 0.413, Supplementary Figure S1A) and LDL-C (0.01 mmol/L, 95% CI −0.16 to 0.17 mmol/L, *p* = 0.941, Figure 3). Total cholesterol and LDL-C concentrations were measured in eleven studies and increased in two studies [21,29], while decreases were observed in three studies [22,28,31]. Ebbeling et al. [26] only measured LDL-C and reported higher concentrations in the LCD group. Increased LDL-C concentrations were found when CHO was solely exchanged for fat, especially SFA [26,29], or when fiber intake decreased [21]. In two studies [22,31], a decrease in LDL-C was found, when CHO was mainly exchanged by protein. Pieke et al. [28] and Vidon et al. [29] both exchanged 14 E% of CHO by comparable amounts of fat (12 and 13 E% respectively) and protein (1.6 and 1.1 E% respectively). With a 10 E% increase in SFA intake, Vidon et al. [29] found an increase in total cholesterol and LDL-C. Pieke et al. [28] reported a significant decrease in total cholesterol by keeping SFA intake comparable between groups and increasing the intake of unsaturated fatty acids (UFAs). De Natale et al. [21] reported significant increases in total cholesterol and LDL-C, when SFA intake was kept constant and UFA intake was increased. However, daily fiber intake decreased by 36 g on the LCD (Supplementary Table S1).

**Figure 4:**
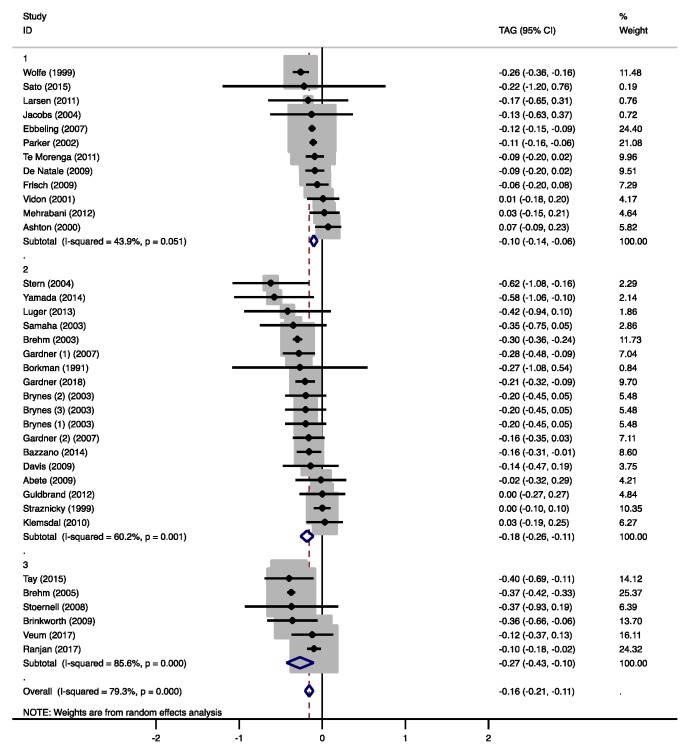
Forest plots of randomized controlled trials that examined the effects of carbohydrate (CHO) restriction on triacylglycerol (TAG) concentrations. Studies were categorized in group 1 (moderate-low CHO, 40–45 E%), group 2 (low CHO, 40–30 E%), and group 3 (very-low CHO, 30–3 E%). Solid squares represent the weight of individual studies and diamonds represent the weighted mean difference (WMD) in TAG. Effects were calculated using random-effect meta-analysis. Decreases in TAGs were significantly lower in the low and very-low CHO group compared to the moderate-low CHO group.

HDL-C concentrations were not significantly affected by the moderate-low CHO diets (0.06 mmol/L, 95% CI −0.05 to 0.15 mmol/L, *p* = 0.326, Supplementary Figure S1B). HDL-C increased in five studies [21,26,28,29,30] that all increased SFA intakes. TAG concentrations were significantly lower compared to the MCDs (−0.10 mmol/L, 95% CI −0.14 to −0.06 mmol/L, *p* < 0.001, Figure 4). In the study of Wolfe et al. [31], TAG concentrations decreased with a 10 E% increase in protein intake, while fat intake remained unchanged. It has been shown that an increase in omega-3 polyunsaturated fatty acids (PUFAs) from fish oil can reduce TAGs [32]. However, only one study that observed lower TAGs with higher PUFA intakes [28] provided information about the marine origin of the PUFAs (Supplementary Table S1).

The moderate-low CHO diets did not affect glucose (0.05 mmol/L, 95% CI −0.22 to 0.31 mmol/L, *p* = 0.730, Supplementary Figure S1C) and insulin concentrations (−0.31 mmol/L, 95% CI −2.61 to 1.98 mmol/L, *p* = 0.789, Supplementary Figure S1D). Glucose concentrations were measured in five studies, but no significant decreases were observed. Vidon et al. [29] only found significant increases in both glucose and insulin concentrations, while another study found a reduction in insulin compared to the MCD [23]. No other studies reported significant changes in insulin concentrations.

In two studies, systolic [25] or diastolic [24] BP decreased significantly. Overall, systolic (−4.05 mmHg, 95% CI −4.99 to −3.10 mmHg, *p* < 0.001, Supplementary Figure S1E). And diastolic (−2.64 mmHg, 95% CI −3.32 to −1.96 mmHg, *p* < 0.001, Supplementary Figure S1F) BP decreased significantly on the moderate-low CHO diets. 

### 3.2. Group 2: Effects of Low CHO Diets (<40–30 E%)

Sixteen studies were identified that compared a low CHO diet (<40–30 E%) to a MCD in different population groups: type 2 diabetes patients [33,34,35,36], patients with at least one characteristic of the metabolic syndrome [37] or one cardiac risk factor [38], overweight or obese participants [39,40,41,42,43] with or without type 2 diabetes [44] and/or metabolic syndrome [45], and healthy volunteers [46,47,48]. Study durations varied between 2 weeks and 24 months. Study characteristics are shown in Table 2.

Compared to the MCDs, overall weight loss in the low CHO group was significantly more pronounced (−1.52 kg, 95% CI −2.92 to −0.12 kg, *p* = 0.034, Figure 2). In five of the 16 studies [35,37,40,41,46], energy restriction was intended in both diet groups. Six studies intended to keep energy intake stable on both diets [34,36,38,39,47,48]. In four studies, a non-energy restricted LCD was compared to an energy restricted diet [33,43,44,45]. In only six studies, the LCD groups showed significant weight losses [40,41,42,43,45,46]. Gardner et al. [42] compared an ad libitum LCD to [1] an energy-restricted MCD and to [2] an isocaloric MCD without energy restriction. Significant weight loss was only observed in the first comparison. After a period of six months, Brehm et al. [43] reported the highest weight loss (6.3 kg), although the reported daily energy intake in the LCD group was 154 kcal higher than in the MCD group. Another study [38] showed a higher energy intake in the LCD groups with no significant effects on body weight after 24 weeks. 

The low CHO diets significantly increased total cholesterol (0.21 mmol/L, 95% CI 0.03 to 0.39 mmol/L, *p* = 0.025, Supplementary Figure S1A), LDL-C (0.11 mmol/L, 95% CI 0.02 to 0.20 mmol/L, *p* = 0.016, Figure 3), and HDL-C concentrations (0.06 mmol/L, 95% CI 0.05 to 0.11 mmol/L, *p* < 0.001, Supplementary Figure S1B). Seven studies, in which SFA intake was increased, found increased HDL-C concentrations [36,39,40,42,44,47,48]. Two of these studies [39,47], in which also fiber intake decreased, also reported higher total cholesterol and/or LDL-C. Gardner et al. [42] compared an LCD with two different MCDs, which resulted in CHO reductions of 9.4 E% and 16 E%. With the highest decrease in CHO intake and the highest increase in fat intake (13 E%), HDL-C concentrations increased significantly. The lowest CHO reduction resulted in significantly lower TAG concentrations. Five other studies found significantly reduced TAGs [39,40,43,44,45], two of which reported increased PUFA intakes of 1.9 E% [40] and 4.1 E% [43]. Gardner et al. [39] increased fat intake by 15 E% and reported a change in fatty-acid composition of +5 E% SFA and of −2 E% monounsaturated fatty acids (MUFA). Although not reported, it can be assumed that PUFA intake increased as well (Supplementary Table S2). Borkman et al. [48] achieved the highest reduction in CHO (−24 E%) as well as protein intakes (−6.1 E%), which were replaced by SFA (15 E%) and MUFA (13 E%) and did not find significant effects on plasma TAGs (Supplementary Table S1). Overall, TAG concentrations significantly decreased in the low CHO group (−0.18 mmol/L, 95% CI −0.26 to −0.11 mmol/L, *p* < 0.001, Figure 4).

Glucose (−0.04 mmol/L, 95% CI −0.19 to 0.10 mmol/L, *p* = 0.556, Supplementary Figure S1C) and insulin concentrations (−0.80 mmol/L, 95% CI −2.06 to 0.46 mmol/L, *p* = 0.212, Supplementary Figure S1D) were not significantly affected by the low CHO diets. Glucose concentrations were measured in 14 studies, but significant effects were only observed in two studies. One study found a significant decrease in plasma glucose, which was accompanied by a reduction in insulin [45], and another study found a significant increase in glucose without any effects on insulin [47]. No further effects on insulin were reported.

Overall effects on systolic (−0.40 mmHg, 95% CI −2.24 to 1.44 mmHg, *p* = 0.671, Supplementary Figure S1E) and diastolic BP (−1.31 mmHg, 95% CI −2.79 to 0.17 mmHg, *p* = 0.082, Supplementary Figure S1F) were not significant in the low CHO group. Straznicky et al. [47] found higher systolic and diastolic BP, while Klemsdaal et al. [37] observed a decrease in diastolic BP. Gardner et al. [42] found significant decreases in systolic BP when CHO intake was reduced by 9.4 E% and decreases in systolic and diastolic BP when CHO intake was decreased by 16 E%.

### 3.3. Group 3: Effects of Very Low-Carbohydrate Diets (<30–3 E%)

Eight studies were identified that assessed the effect of a very low-carbohydrate diet (<30–3 E%) compared to a MCD in type 2 diabetes patients [49,50], overweight or obese participants [51], patients with abdominal obesity [52] and at least one other metabolic syndrome risk factor [53], participants with elevated TAGs [54], and healthy men [55,56]. Study durations varied between 1 week and 12 months. Study characteristics are shown in Table 3.

Overall weight loss was significantly higher with the very-low CHO diets compared to the MCDs (−1.62 kg, 95% CI −2.64 to −0.60 kg, *p* = 0.002, Figure 2). In two studies [50,53], energy restriction was intended in both diet groups, while five studies intended to keep energy intake stable on both diets [49,52,54,55,56]. Two studies reported significant weight losses [51,55] without reductions in reported energy intakes. In one of these studies [51], the aim was to compare a non-energy restricted LCD to an energy-restricted MCD. 

Total cholesterol was not significantly affected by the very-low CHO diets (0.26 mmol/L, 95% CI −0.02 to 0.54 mmol/L, *p* = 0.074, Supplementary Figure S1A), while LDL-C (0.32 mmol/L, 95% CI 0.08 to 0.56 mmol/L, *p* = 0.010, Figure 3) and HDL-C concentrations (0.13 mmol/L, 95% CI 0.11 to 0.16 mmol/L, *p* < 0.001, Supplementary Figure S1B) were significantly higher. In two studies, total cholesterol, LDL-C, and HDL-C significantly increased with higher SFA and dietary cholesterol intakes in the very-low CHO group [52,53]. Brehm et al. [51] only found increases in HDL-C, but no information on the fatty acid composition of the LCD was given (Supplementary Table S3). Tay et al. [50] replaced CHO mainly by UFAs and proteins, and found increased HDL-C concentrations, as well as decreased TAG concentrations. In contrast, Brinkworth et al. [53] reported significantly lower TAGs with a more pronounced decrease in CHO intake, but much higher increases in SFA and lower increases in PUFA intakes (Supplementary Table S3). Overall, TAG concentrations significantly decreased in the very-low CHO group (−0.27 mmol/L, 95% CI −0.43 to −0.10 mmol/L, *p* = 0.001, Figure 4).

Glucose was not significantly affected by the very-low CHO diets (0.08 mmol/L, 95% CI −0.11 to 0.26 mmol/L, *p* = 0.406, Supplementary Figure S1C), while insulin was significantly lower (−0.86 mmol/L, 95% CI −1.61 to −0.11 mmol/L, *p* = 0.025, Supplementary Figure S1D). One study [52] found significantly increased plasma glucose levels, while insulin was not significantly affected in any of the studies. 

No effects on systolic (−0.21 mmHg, 95% CI −3.06 to 2.63 mmHg, *p* = 0.884, Supplementary Figure S1E) and diastolic BP (1.53 mmHg, 95% CI −1.20 to 4.25 mmHg, *p* = 0.271, Supplementary Figure S1F) were detected. 

### 3.4. Effects of the Degree of CHO Restriction

#### 3.4.1. Weight

Combining the results of all LCD groups, a significant weight loss (−1.34 kg, 95% CI −2.26 to −0.42 kg, *p* = 0.004) was observed. Differences between the groups did not however reach statistical significance (group 2 vs. 1: −0.89 kg, 95% CI −2.35 to 0.58 kg, *p* = 0.226; group 3 vs. 1: −0.95 kg, 95% CI −2.72 to 0.82 kg, *p* = 0.280, Figure 2 and Supplementary Figure S2A). A meta-analysis by Johnston et al. [57] also found that significant weight loss was found with any low-carbohydrate diet and that weight loss differences between the diets were small. In most studies, weight loss was due to a lower energy intake, although energy intake was often not explicitly restricted in the LCD groups. The unintended reduction in energy intake can be explained by the satiating effect of fat and protein, an appetite-suppressing effect of ketones, as well as increased attention to dietary behavior [58,59]. However, not all studies that showed a significant weight loss effect of the LCD reported a lower energy intake compared to the MCD. The authors explained these findings by unreliable food records, or an increased energy expenditure caused by physical activity or possible thermogenic effects of the LCD [43,51]. 

#### 3.4.2. Total Cholesterol and LDL-Cholesterol

Overall, a significant increase in total cholesterol was observed with the LCDs (0.10 mmol/L, 95% CI 0.01 to 0.18 mmol/L, *p* = 0.029). Differences between the three CHO groups showed a trend towards statistical significance with lower total cholesterol levels in group 1 compared to the other two groups (group 2 vs. 1: 0.28 mmol/L, 95% CI −0.02 to 0.57 mmol/L, *p* = 0.066; group 3 vs. 1: 0.30 mmol/L, 95% CI −0.05 to 0.65 mmol/L, *p* = 0.086, Supplementary Figures S1A and S2B). 

Compared to the MCDs, significantly higher LDL-C concentrations were observed with the LCDs (0.10 mmol/L, 95% CI 0.02 to 0.17 mmol/L, *p* = 0.009). These effects were significantly different between the three CHO groups with more pronounced increases in the very-low CHO diet compared to the moderate-low CHO diet (group 3 vs. 1: 0.35 mmol/L, 95% CI 0.07 to 0.62 mmol/L, *p* = 0.015, Figure 3 and Supplementary Figure S2C). Differences between the moderate-low and the low CHO group did not reach statistical significance (group 2 vs. 1: 0.13 mmol/L, 95% CI −0.08 to 0.33 mmol/L, *p* = 0.215). It is well-known that SFA [60], dietary cholesterol [61], as well as fiber intakes [13] affect plasma total and LDL-C concentrations. In this review, the studies that found increased total cholesterol and LDL-C concentrations reported increases in SFA intakes, which were often accompanied by increased dietary cholesterol or decreased fiber intakes (Supplementary Tables S1–S3).

#### 3.4.3. HDL Cholesterol

Overall, HDL-C was significantly increased after the LCD interventions (0.08 mmol/L, 95% CI 0.04 to 0.13 mmol/L, *p* < 0.001). However, no significant differences were detected between the three CHO groups (group 2 vs. 1: 0.05 mmol/L, 95% CI −0.02 to 0.12 mmol/L, *p* = 0.173; group 3 vs. 1: 0.08 mmol/L, 95% CI −0.01 to 0.18 mmol/L, *p* = 0.070, Supplementary Figures S1B and S2D). The studies that found significant increases in HDL-C, all increased total fat intake in the form of SFAs or UFAs, or a combination of both (Supplementary Tables S1–S3). Previous research has shown that replacement of CHO for SFAs or UFAs increases HDL-C concentrations [14]. In addition, higher-protein diets are associated with higher HDL-C [62]. In this review, ten of the 15 studies that found increased HDL-C concentrations also reported increased protein intakes. However, it remains uncertain whether increased HDL-C concentrations per se have favorable effects on CVD risk [63], and the unfavorable changes in LDL-C should be considered as well [31,32].

#### 3.4.4. Triacylglycerol

Overall, TAG concentrations significantly decreased with the LCDs (−0.16 mmol/L, 95% CI −0.21 to −0.11 mmol/L, *p* < 0.001). Based on earlier studies [14,58], it was expected that a more pronounced CHO reduction would result in lower TAG concentrations. Indeed, significant differences between the CHO groups were detected (group 2 vs. 1: −0.13, 95% CI −0.25 to −0.01 mmol/L, *p* = 0.028; group 3 vs. 1: −0.19, 95% CI −0.35 to −0.04 mmol/L, *p* = 0.017, Figure 4 and Supplementary Figure S2E). The studies that showed beneficial effects on TAG concentrations and provided information on the fatty-acid composition, also reported an increase in PUFA intake (Supplementary Table S1). 

#### 3.4.5. Glucose and Insulin

For glucose (0.00 mmol/L, 95% CI −0.09 to 0.10 mmol/L, *p* = 0.921) and insulin (−0.60 μU/mL, 95% CI −1.46 to 0.26 μU/mL, *p* = 0.169), no overall significant effects were observed. There were also no significant differences between the CHO groups for glucose (group 2 vs. 1: −0.28 mmol/L, 95% CI −0.99 to 0.43 mmol/L, *p* = 0.420; group 3 vs. 1: −0.02, 95% CI −0.83 to 0.79 mmol/L, *p* = 0.961, Supplementary Figures S1C and S2F) and insulin (group 2 vs. 1: 0.09 μU/mL, 95% CI −3.90 to 4.08 μU/mL, *p* = 0.961; group 3 vs. 1: −0.02 μU/mL, 95% CI −4.95 to 4.91 μU/mL, *p* = 0.993, Supplementary Figures S1D and S2G). 

LCDs resulted in increased glucose concentrations when compared to MCDs with a higher intake of fiber [47,52]. An increased fiber intake has been shown to improve glycemic control, insulin sensitivity, and plasma glucose levels [64]. A systematic review by Churuangsuk et al. [65] identified five meta-analyses that did not find significant effects of LCDs on glucose, as well as three meta-analyses without significant effects on insulin. The meta-analysis by Naude et al. [66] performed a subgroup analysis in diabetic and non-diabetic participants without finding significant effects on glucose concentrations in neither group.

#### 3.4.6. Blood Pressure

For systolic BP (−1.1 mmHg, 95% CI −2.5 to 0.3 mmHg, *p* = 0.132), no overall significant effects were observed, whereas an overall significant reduction in diastolic BP (−1.1 mmHg, 95% CI −2.1 to −0.1 mmHg, *p* = 0.040) was found. The changes in systolic BP (group 2 vs. 1: 1.9 mmHg, 95% CI ‒2.2 to 6.0 mmHg, *p* = 0.354; group 3 vs. 1: 1.3 mmHg, 95% CI −3.7 to 6.4, *p* = 0.588 mmHg, Supplementary Figures S1E and S3A) and diastolic BP (group 2 vs. 1: 0.6 mmHg, 95% CI −2.1 to 3.4 mmHg, *p* = 0.635; group 3 vs. 1: 2.5 mmHg, 95% CI −0.9 to 5.9 mmHg, *p* = 0.138, Supplementary Figures S1F and S3B) did not depend on the degree of CHO restriction. It should be noted that in the majority of studies that reported BP, the LCD and the MCD both resulted in lower BP, without significant differences between the diets. It remains uncertain whether both diets were evenly effective, or whether BP decreased in both groups due to an acclimatization of the participants to the study environment. Other meta-analyses comparing LCDs with LFDs either found no significant differences between the diets in systolic and diastolic BP [66,67], or a more pronounced decrease in diastolic BP on the LCD [68,69].

### 3.5. Subgroup Analysis

Subgroup analyses suggested that health status (healthy, overweight/obese, or metabolically impaired) was a source of heterogeneity (Supplementary Table S4). Weight loss was significantly larger in overweight or obese compared to healthy participants (*p* = 0.039). In the metabolically impaired populations, increases in LDL-C were less pronounced compared to healthy participants (*p* = 0.033). Larger decreases in systolic BP were observed in the overweight or obese group compared to the healthy participants (*p* = 0.015). 

Changes in weight loss were identified as a source of heterogeneity for TAG concentrations in the low CHO group, showing more pronounced decreases in the subgroup with higher weight loss (*p* = 0.036, overall effects are shown in Supplementary Table S5). In the subgroup with higher SFA intakes, higher overall increases in total cholesterol (*p* = 0.011), LDL-C (*p* = 0.015), and HDL-C (*p* = 0.037) concentrations were observed (Supplementary Table S6). Protein intake was a source of heterogeneity for overall LDL-C (*p* = 0.038) and glucose (*p* = 0.042) concentrations, as shown by less pronounced increases in the subgroups with an increased protein intake (Supplementary Table S7). The study design (parallel vs. cross-over) was related to overall weight loss (*p* = 0.021, Supplementary Table S8) with higher weight loss in parallel-designed studies. The study design also affected TAG concentrations in the very-low CHO group with less pronounced decreases in the cross-over designed studies (*p* = 0.038, data not shown). Study duration was associated with total cholesterol, LDL-C, and HDL-C concentrations in the low CHO group, as shown by more pronounced decreases in studies with durations exceeding one month (total cholesterol *p* < 0.001, LDL *p* < 0.001, HDL *p* = 0.007) and six months (total cholesterol *p* < 0.001, LDL *p* < 0.001, HDL *p* = 0.014), compared to studies with durations of less than one month (overall effects are shown in Supplementary Table S9).

## 4. Limitations

In this systematic review and meta-analysis, we compared the effects of different degrees of CHO reduction on cardiometabolic risk markers, and interpreted our findings by analyzing and discussing potential effects of the nutrients used for CHO substitution. One limitation of our study is that hardly any information was available on the different types of SFAs or PUFAs, which are known to have different metabolic effects [59]. In addition, the type of CHO, the glycemic index (GI), or the glycemic load (GL) of the intervention diets were often not described into detail. These limitations may have been additional sources of heterogeneity that could not be investigated. 

Although non-fasting measurements, including the assessment of postprandial dyslipidemia and glycaemia, provide important information on metabolic health and CVD risk, the majorities of studies only reported fasting measurements. Postprandial TAGs have been independently associated with the incident of cardiovascular events [70], while increased postprandial glycemia is related to an increased risk of obesity, type 2 diabetes mellitus, and the metabolic syndrome [71]. The addition of non-fasting measurements is therefore important to fully assess the potential health benefits of dietary interventions.

Due to the large age diversity of the study populations between and within selected studies, and due to the inclusion of both men and women in the majority of studies, subgroup analyses to assess the effect of age and gender on cardiometabolic risk markers were not considered feasible. We recommend performing these subgroup analyses in future studies.

## 5. Conclusions

This meta-analysis showed that the LCDs resulted in small—but statistically significant—effects on weight loss, increases in HDL-C, and decreases in TAG concentrations and diastolic BP, compared to the MCDs. Decreases in TAG concentrations were more pronounced with lower CHO intakes. However, a higher degree of CHO restriction did not provide additional health benefits on the other CVD risk markers. In fact, LDL-C concentrations were the highest on the very-low CHO diets (<30–3 E%). These increases could be explained by higher intakes of SFA as replacement for CHO, as well as a decreased fiber intake which often accompanies CHO reduction. Therefore, our results suggest that potential benefits and risks of LCDs are rather related to CHO replacement than CHO restriction per se. Future studies should therefore also focus on the types of foods used as CHO substitute.

## Figures and Tables

**Table 1:** Overview of the moderate low-carbohydrate diets (<45–40 E%) and their effects on cardiometabolic risk markers.

Study Characteristics					Diet Changes				Diet Effect							
Author And Year	Study Design	LCD vs. MCD	Duration	Participants	kcal	CHO	Fat	Prot	Weight (kg)	TC (mmol/L)	LDL−C	HDL−C	TAG	Glucose (mmol/L)	Insulin (mU/mL)	Systolic/
											(mmol/L)	(mmol/L)	(mmol/L)			Diastolic BP (mmHg)
Sato [19]	Parallel	Isocaloric	6 months	62 participants with T2D	−277	−7.3	5.6	2.7	↓ −1.0		=	=	=			
2017																
Larsen [20] 2011	Parallel	Isocaloric with ER	12 months	99 participants with T2D	75	−5.2	−0.7	6.6	=	=	=	=	=			=/=
Parker [22] 2002	Parallel	Isocaloric with ER	3 months	46 participants with T2D	244	−12	0.9	12	=	↓ −0.3	↓ −0.3	=	=	=	=	=/=
Mehrabani [23] 2012	Parallel	Isocaloric	12 weeks	49 overweight/	−143	−11	−3.1	15	=	=	=	=	=		↓ −2.6	
				obese women with PCOS												
Te Morenga [24] 2011	Parallel	Isocaloric with ER	2 months	74 overweight/	214	−9	4	6	↓ −1.3	=	=	=	=	=	=	=/
				obese women												↓ −3.7
Frisch [25] 2009	Parallel	Isocaloric with ER	12 months	200 overweight participants	−64	−4.3	2.5	1.4	=	=	=	=	=	=		↓ −4.0/=
Ebbeling [26] 2007	Parallel	Isocaloric	18 months	73 obese participants	154	−13	12	−0.3	=		↑ 0.3	↑ 0.3	=	=	=	=/=
De Natale [21] 2009	Cross−	Isocaloric	4 weeks	18 participants with T2D	−12	−7	7	0	=	↑ 0.2	↑ 0.2	↑ 0.1	=			
	over															
Jacobs [27] 2004	Cross−over	Isocaloric	3 weeks	14 non−obese men with elevated TAGs	48	−11	11	−0.3	=	=	=	=	=			
Pieke [28] 2000	Cross−over	Isocaloric	3 weeks	19 non−obese men with elevated TAGs	81	−14	12	1.6		↓ −0.2	=	↑ 0.1	↓ −0.9		=	
Vidon [29] 2001	Cross−over	Isocaloric	3 weeks	7 healthy participants		−14	13	1.1		↑ 0.6	↑ 0.4	↑ 0.2	=	↑ 0.6	↑ 2.6	
Ashton [30] 2000	Cross−over	Isocaloric	3 weeks	28 healthy participants	300	−14	16	0.8	=	=	=	↓ −0.1	=			=/=
Wolfe [31] 1999	Cross−over	Isocaloric	4 weeks	10 healthy participants	23	−10	0	10	=	↓ −0.3	↓ −0.2	=	↓ −0.3			

Diet changes and diet effects have been calculated as differences between the low-carbohydrate diet (<45–40 E% from CHO) and the moderate-carbohydrate diet (45–55 E% from CHO). ↑ or ↓ or =, indicates a statistically significant (*p* < 0.05) increase (↑) or decrease (↓) or no difference ( = ). Abbreviations: E%, percent of total energy; LCD, low-carbohydrate diet; MCD, moderate-carbohydrate diet; CHO, carbohydrate; Prot, Protein; TC, total cholesterol; LDL-C: low-density lipoprotein cholesterol; HDL-C, high-density lipoprotein cholesterol; TAG, triacylglycerol; BP, blood pressure; T2DM, type 2 diabetes mellitus; ER, energy restriction.

**Table 2:** Overview of the low-carbohydrate diets (<40–30 E%) and their effects on cardiometabolic risk markers.

Study Characteristics					Diet Changes				Diet Effect							
Author And Year	Study Design	LCD vs. MCD	Duration	Participants	kcal	CHO	Fat	Prot	Weight (kg)	TC (mmol/L)	LDL−C	HDL−C	TAG	Glucose (mmol/L)	Insulin (mU/mL)	Systolic/
											(mmol/L)	(mmol/L)	(mmol/L)			Diastolic BP (mmHg)
Yamada [33] 2014	Parallel	*Ad libitum* vs. ER	6 months	24 participants with T2D	24	−21	13	8.7	=		=	=	=	=		=/=
Luger [34]	Parallel	Isocaloric	12 weeks	42 participants with T2D	44	−13	6.7	5.9	=		=	=	=	=		=/=
2013																
Guldbrand [35] 2012	Parallel	Isocaloric with ER	24 months	61 participants with T2D	−189	−9	6	4	=	=	=	=	=			=/=
Davis [36]	Parallel	Isocaloric	12 months	105 participants with T2D	−288	−19	16	3.7	=	=	=	↑ 0.1	=			=/=
2009																
Klemsdal [37] 2010	Parallel	Isocaloric with ER	12 months	164 participants with at least one MetS symptom		−8.1	5	2.5	=	=	=	=	=	=	=	=/
																↓ −2.9
Gardner [39] 2018	Parallel	Isocaloric	12 months	609 overweight participants	−94	−18	15	1.9	=		↑ 0.2	↑ 0.1	↓ −0.2	=	=	=/=
Bazzano [40] 2014	Parallel	Isocaloric with ER	12 months	148 obese participants	−43	−22	13	5.3	↓ −3.5	=	=	↑ 0.2	↓ −0.2	=	=	=/=
Abete [41] 2009	Parallel	Isocaloric with ER	8 weeks	19 obese men		−19	4.2	11	↓ −3.2	=	=	=	=	=	=	=/=
Gardner [42] 2007 (1)	Parallel	*Ad libitum* vs. ER	12 months	311 overwight/obse woman	92	−9.4	9.2	−0.4	↓ −3.1		**=**	**=**	**↓** −0.3	**=**	**=**	↓ −4.3/=
Gardner [42] 2007 (2)	Parallel	Isocaloric	12 months	311 overwight/obse woman	56	−16	13	2.0	**=**		=	↑ 0.2	**=**	=	=	↓ −5.7/↓ −3.7
Brehm [43] 2003	Parallel	*Ad libitum* vs. ER	6 months	42 obese men	154	−23	18	4.0	↓ −6.3	=	=	=	↓ −0.3	=	=	=/=
Stern [44] 2004	Parallel	*Ad libitum* vs. ER	12 months	87 obse participants +/− T2D	−413	−16	25	0.7	=	=	=	↑ 0.1	↓ −0.6			=/=
Samaha [45] 2003	Parallel	*Ad libitum* vs. ER	6 months	132 obse participants +/− T2D and MetS	−188	−12	8.0	5.0	↓ −3.9	=	=	=	↓ −0.4	↓ −0.5	↓ −7.0	=/=
Hu [46]	Parallel	Isocaloric with ER	12 months	148 healthy participants	−43	−22	13	5.3	↓ −3.5							
2015																
Brynes [38] 2003 (1)	Cross−over	Isocaloric	24 weeks	17 men with at least one cardiac risk factor	449	−11	11	−2.0	=	=	=	=	=	=	=	
Brynes [38] 2003 (2)	Cross−over	Isocaloric	24 weeks	17 men with at least one cardiac risk factor	736	−11	14	−3.0	=	=	=	=	=	=	=	
Brynes [38] 2003 (3)	Cross−over	Isocaloric	24 weeks	17 men with at least one cardiac risk factor	239	−15	14	−1.0	=	=	=	=	=	=	=	
Straznicky [47] 1999	Cross−over	Isocaloric	2 weeks	148 healthy men	449	−18	22	−2.9	=	↑ 1.1	↑ 0.9	↑ 0.2	=	↑ 0.3	**=**	↑ 7.0/ ↑ 3.0
Borkman [48] 1991	Cross−over	Isocaloric	3 weeks	8 healthy participants	287	−24	30	−6.1	=	=	=	↑ 0.3	=			

Diet changes and diet effects have been calculated as differences between the low-carbohydrate diet (<40–30 E% from CHO) and the moderate-carbohydrate diet (45–55 E% from CHO). ↑ or ↓ or =, indicates a statistically significant (*p* < 0.05) increase (↑) or decrease (↓) or no difference ( = ). Abbreviations: E%, percent of total energy; LCD, low-carbohydrate diet; MCD, moderate-carbohydrate diet; CHO, carbohydrate; Prot, Protein; TC, total cholesterol; LDL-C: low-density lipoprotein cholesterol; HDL-C, high-density lipoprotein cholesterol; TAG, triacylglycerol; BP, blood pressure; ER, energy restriction; T2DM, type 2 diabetes mellitus; MetS, metabolic syndrome.

**Table 3:** Overview of the very low-carbohydrate diets (<30–3 E%) and their effects on cardiometabolic risk markers.

Study Characteristics					Diet Changes				Diet Effect							
Author And Year	Study Design	LCD vs. MCD	Duration	Participants	kcal	CHO	Fat	Prot	Weight (kg)	TC (mmol/L)	LDL−C (mmol/L)	HDL−C (mmol/L)	TAG (mmol/L)	Glucose (mmol/L)	Insulin (μU/mL)	Systolic/ Diastolic BP (mmHg)
Tay [50] 2015	Parallel	Isocaloric with ER	12 months	77 participants with T2D	−37	−32	26	7.2		=	=	↑ 0.1	↓ −0.4	=	=	=/=
Brehm [51] 2005	Parallel	*Ad libitum* vs. ER	4 months	40 obese women	119	−21	19	2.0	↓ −3.7	=	=	↑ 0.1	=			=/=
Veum [52] 2017	Parallel	Isocaloric	12 weeks	38 men with abdominal obesity	245	−39	40	−0.6	=	↑ 1.1	↑ 1.0	↑ 0.2	=	↑ 0.3	=	=/=
Brinkworth [53] 2009	Parallel	Isocaloric with ER	12 months	107 participants with abdominal obesity and at least one MetS symptom	20	−38	29	11	=	↑ 0.6	↑ 0.6	↑ 0.2	↓ −0.4	=	=	=/=
Stoernell [54] 2008	Parallel	Isocaloric	8 weeks	23 participants with elevated TAGs	−145	−27	21	2.0	=	=	=	=	=			
Ranjan [49] 2017	Cross− over	Isocaloric	1 week	10 participants with T2D	−160	−40	31	9.2	=	=	=	=	=	=		=/=
Holloway [55] 2011	Cross− over	Isocaloric	5 days	16 healthy men	−31	−45	47	−2.0	↓ −3.1	=				=		
Chokkalingam [56] 2007	Cross− over	Isocaloric	6 days	10 healthy men	215	−42	44	0						=	=	

Diet changes and diet effects have been calculated as differences between the low-carbohydrate diet (<30–3 E% from CHO) and the moderate-carbohydrate diet (45–55 E% from CHO). ↑ or ↓ or =, indicates a statistically significant (*p* < 0.05) increase (↑) or decrease (↓) or no difference ( = ). Abbreviations: E%, percent of total energy; LCD, low-carbohydrate diet; MCD, moderate-carbohydrate diet; CHO, carbohydrate; Prot, Protein; TC, total cholesterol; LDL-C: low-density lipoprotein cholesterol; HDL-C, high-density lipoprotein cholesterol; TAG, triacylglycerol; BP, blood pressure; ER, energy restriction; T2DM, type 2 diabetes mellitus; MetS, metabolic syndrome.

## Supplementary Materials

The following are available online at https://www.mdpi.com/2072-6643/12/4/991/s1. Table S1. Changes in the compositions of the moderate low-carbohydrate diets (<45–40 E%), Table S2. Changes in the compositions of the low-carbohydrate diets (<40–30 E%), Table S3. Changes in the compositions of the very low-carbohydrate diets (<30–3 E%), Table S4. Effect of health status on cardiovascular disease (CVD) risk markers, Table S5. Effect of weight loss on CVD risk markers, Table S6. Effect of SFA-intake on CVD risk markers, Table S7. Effect of protein-intake on CVD risk markers, Table S8. Effect of study design on CVD risk markers, Table S9. Effect of study duration on CVD risk markers, Figure S1A. Forest plots of randomized controlled trials that examined the effects of CHO restriction on total cholesterol concentrations, Figure S1B. Forest plots of randomized controlled trials that examined the effects of CHO restriction on HDL-C concentrations, Figure S1C. Forest plots of randomized controlled trials that examined the effects of CHO restriction on plasma glucose concentrations, Figure S1D. Forest plots of randomized controlled trials that examined the effects of CHO restriction on serum insulin concentrations, Figure S1E. Forest plots of randomized controlled trials that examined the effects of CHO restriction on systolic blood pressure, Figure S1F. Forest plots of randomized controlled trials that examined the effects of CHO restriction on diastolic blood pressure, Figure S2. Comparison of the diet effects of the moderate-low (<45–40 E%), low (<40–30 E%), and very low (<30–3 E%) carbohydrate groups on (A) weight, (B) total cholesterol, (C) LDL-cholesterol, (D) HDL-cholesterol, (E) triacylglycerol, (F) glucose, and (G) insulin, Figure S3. Comparison of the diet effects of the moderate low (<45–40 E%), low (<40–30 E%), and very low (<30–3 E%) carbohydrate groups on (A) systolic and (B) diastolic blood pressure.
