# Supplementary material for: The Effects of Different Degrees of Carbohydrate Restriction and Carbohydrate Replacement on Cardiometabolic Risk Markers in Humans—A Systematic Review and Meta-Analysis
Source: Nutrients. 2020 Apr 2;12(4):991. doi: 10.3390/nu12040991 (PMC7230871; doi:10.3390/nu12040991)
Supplement: Supplementary file 1 [file nutrients-12-00991-s001.pdf]

Table S1. Changes in the compositions of the moderate low-carbohydrate diets (<45–40 E%).

Author and year	LCD vs. MCD	Energy (kcal)	CHO (%)	Fiber (g)	Fat (%)	SFA (%)	MUFA (%)	PUFA (%)	Protein (%)	Chol (mg)
Sato (19) 2017	Isocaloric	-277	-7.3		5.6	3.2	0.8	0.7	2.7	
Larsen (20) 2011	Isocaloric with ER	75	-5.2	-1.4	-0.7	-0.9	0	-0.3	6.6	53
Parker (22) 2002	Isocaloric with ER	244	-12	-4.2	0.9	0.6	0.6	-0.1	12	104
Mehrabani (23) 2012	Isocaloric	-143	-11		-3.1				15	
Te Morenga (24) 2011	Isocaloric with ER	214	-9.0	-14	4.0	3.0			6.0	
Frisch (25) 2009	Isocaloric with ER	-64	-4.3		2.5				1.4	
Ebbeling (26) 2007	Isocaloric	154	-13	-2.0	12	4.8			-0.3	
De Natale (21) 2009	Isocaloric	-12	-7.0	-36	7.0	0	6.0	0.1	0	27
Jacobs (27) 2004	Isocaloric	48	-11		11	3.5	5.0	-2.1	-0.3	15
Pieke (28) 2000	Isocaloric	81	-14	-1.2	12	0.9	5.0	4.7	1.6	29
Vidon (29) 2001	Isocaloric		-14		13				1.1	21
Ashton (30) 2000	Isocaloric	300	-14	0.6	16	0.6	16	-0.4	0.8	-8.0
Wolfe (31) 1999	Isocaloric	23	-10	-2.1	0				10	1.0

Abbreviations: LCD, low carbohydrate diet; MCD, moderate carbohydrate diet; CHO, carbohydrate; SFA, saturated fatty acid; Chol, cholesterol; ER, energy restriction.

Table S2. Changes in the compositions of the low-carbohydrate diets (<40–30 E%).

Author and year	LCD vs. MCD	Energy (kcal)	CHO (%)	Fiber (g)	Fat (%)	SFA (%)	MUFA (%)	PUFA (%)	Protein (%)	Chol (mg)
Yamada (32) 2014	*Ad libitum* vs. ER	24	-21		13				8.7	
Luger (33) 2013	Isocaloric	44	-13	3.3	6.7				5.9	
Guldbrand (34) 2012	Isocaloric with ER	-189	-9.0		6.0	3.0	3.1	1.9	4.0	
Davis (35) 2009	Isocaloric	-288	-19	-2.6	16	1.4	4.1	-0.2	3.7	
Klemsdal (36) 2010	Isocaloric with ER		-8.1		5				2.5	
Gardner (38) 2018	Isocaloric	-94	-18	-4.4	15	5.1	-2.1		1.9	
Bazzano (39) 2014	Isocaloric with ER	-43	-22	-2.1	13	5.5	4.7	1.9	5.3	0
Abete (40) 2009	Isocaloric with ER		-19	-4.5	4.2	9.3	-2.3	0.4	11	257
Gardner (41) 2007 [1]	*Ad libitum* vs. ER	92	-9.4	-1.1	9.2	2.8			-0.4	
Gardner (41) 2007 [2]	Isocaloric	56	-16	-4.9	13	4.6			2.0	
Brehm (42) 2003	*Ad libitum* vs. ER	154	-23	-3.5	18	6.2	8.5	4.1	4.0	162

Stern (43) 2004	*Ad libitum* vs. ER	-413	-16		25	3.6			0.7	
Samaha (44) 2003	*Ad libitum* vs. ER	-188	-12		8.0				5.0	
Hu (45) 2015	Isocaloric with ER	-43	-22	-2.3	13	5.5	4.7		5.3	
Brynes (37) 2003 [1]	Isocaloric	449	-11	3.0	11		6.1		-2.0	
Brynes (37) 2003 [2]	Isocaloric	736	-11	-9.0	14		7.1		-3.0	
Brynes (37) 2003 [3]	Isocaloric	239	-15	3.0	14		7.1		-1.0	
Straznicky (46) 1999	Isocaloric	449	-18	-11	22	19	8.3	-5.8	-2.9	343
Borkman (47) 1991	Isocaloric	287	-24	-23	30	15	13	1.4	-6.1	280

Abbreviations: LCD, low carbohydrate diet; MCD, moderate carbohydrate diet; CHO, carbohydrate; SFA, saturated fatty acid; Chol, cholesterol; ER, energy restriction.

Table S3. Changes in the compositions of the very low-carbohydrate diets (<30–3 E%).

Author and year	LCD vs. MCD	Energy (kcal)	CHO (%)	Fiber (g)	Fat (%)	SFA (%)	MUFA (%)	PUFA (%)	Protein (%)	Chol (mg)
Tay (49) 2015	Isocaloric with ER	-37	-32	-5.7	26	2.5	17	6.9	7.2	119
Brehm (50) 2005	*Ad libitum* vs. ER	119	-21		19				2.0	
Veum (51) 2017	Isocaloric	245	-39	-12	40	19	13	0.5	-0.6	371
Brinkworth (52) 2009	Isocaloric with ER	20	-38		29	14	12	1.2	11	443
Stoernell (53) 2008	Isocaloric	-145	-27		21	3.0			2.0	
Ranjan (48) 2017	Isocaloric	-160	-40		31				9.2	
Holloway (54) 2011	Isocaloric	-31	-45		47				-2.0	
Chokkalingam (55) 2007	Isocaloric	215	-42		44				0	

Abbreviations: LCD, low carbohydrate diet; MCD, moderate carbohydrate diet; CHO, carbohydrate; SFA, saturated fatty acid; Chol, cholesterol; ER, energy restriction.

Table S4. Effect of health status on cardiovascular disease (CVD) risk markers.

Risk marker	Categories *	Difference WMD ± SEM	Lower 95% CI	Upper 95% CI	p-value
Weight (kg)	Overweight/obese	-1.82 ± 0.84	-3.53	-0.10	0.039
	Metab. impaired	-0.30 ± 0.73	-1.80	1.20	0.685
Total chol. (mmol/l)	Overweight/obese	-0.27 ± 0.19	-0.67	0.12	0.172
	Metab. impaired	-0.32 ± 0.17	-0.67	0.03	0.072
LDL-C (mmol/l)	Overweight/obese	0.22 ± 0.15	-0.52	0.08	0.144
	Metab. impaired	-0.31 ± 0.14	-0.59	-0.03	0.033
HDL-C (mmol/l)	Overweight/obese	-0.05 ± 0.05	-0.15	0.06	0.367
	Metab. impaired	-0.06 ± 0.05	-0.15	0.04	0.254
TAG (mmol/l)	Overweight/obese	-0.07 ± 0.09	-0.23	0.11	0.444
	Metab. impaired	-0.15 ± 0.08	-0.32	0.01	0.064
Glucose (mmol/l)	Overweight/obese	-0.19 ± 0.32	-0.86	0.47	0.556
	Metab. impaired	-0.56 ± 0.31	-1.21	0.08	0.083
Insulin (µU/ml)	Overweight/obese	0.09 ± 1.90	-3.90	4.08	0.961
	Metab. impaired	-0.02 ± 2.35	-4.95	4.91	0.993
Systolic BP (mmHg)	Overweight/obese	-6.98 ± 2.62	-12.4	-1.53	0.015
	Metab. impaired	-3.90 ± 2.62	-9.36	1.55	0.152
Diastolic BP (mmHg)	Overweight/obese	-2.33 ± 2.14	-6.78	2.12	0.288

	Metab. impaired	-1.67 ± 2.14	-6.12	2.78	0.443

* Changes in overweight/obese and metabolically impaired participants were compared to those in healthy participants as reference category. Abbreviations: WMD, weighted mean differences; Metab., metabolically.

Table S5. Effect of changes in weight loss on cardiovascular disease (CVD) risk markers *.

Risk marker	Difference WMD ± SEM	Lower 95% CI	Upper 95% CI	*p*-value
Total chol. (mmol/L)	0.08 ± 0.15	-0.22	0.38	0.597
LDL-C (mmol/L)	0.04 ± 0.11	-0.18	0.26	0.714
HDL-C (mmol/L)	-0.02 ± 0.03	-0.08	0.05	0.573
TAG (mmol/L)	0.10 ± 0.06	-0.02	0.22	0.094
Glucose (mmol/L)	0.24 ± 0.15	-0.06	0.55	0.114
Insulin (µU/mL)	0.76 ± 1.52	-2.53	4.05	0.626
Systolic BP (mmHg)	2.79 ± 1.46	-0.28	5.85	0.072
Diastolic BP (mmHg)	2.42 ± 1.18	-0.05	4.88	0.054

* The median change in weight loss (–0.85 kg) was used as stratification variable to create two subgroups (subgroup 1 ≤ median change vs. subgroup 2 > median change). Abbreviations: WMD, weighted mean differences.

Table S6. Effect of changes in saturated fatty acid (SFA) intake on cardiovascular disease (CVD) risk markers*.

Risk marker	Difference WMD ± SEM	Lower 95% CI	Upper 95% CI	p-value
Weight (kg)	-1.62 ± 0.84	-3.39	0.14	0.069
Total chol. (mmol/L)	0.50 ± 0.18	0.13	0.87	0.011
LDL-C (mmol/L)	0.29 ± 0.11	0.06	0.52	0.015
HDL-C (mmol/L)	0.09 ± 0.04	0.01	0.17	0.037
TAG (mmol/L)	-0.09 ± 0.05	-0.19	0.02	0.107
Glucose (mmol/L)	-0.10 ± 0.16	-0.46	0.26	0.558
Insulin (µU/mL)	-0.07 ± 1.33	-2.97	2.84	0.961
Systolic BP (mmHg)	0.50 ±1.92	-3.56	4.57	0.796
Diastolic BP (mmHg)	0.83 ± 1.43	-2.19	3.86	0.566

* The median change in SFA intake (3.5 E%) was used as stratification variable to create two subgroups (subgroup 1 ≤ median change vs. subgroup 2 > median change). Abbreviations: WMD, weighted mean differences.

Table S7. Effect of changes in protein-intake on cardiovascular disease (CVD) risk markers *.

Risk marker	Difference WMD ± SEM	Lower 95% CI	Upper 95% CI	*p*-value
Weight (kg)	-1.01 ± 0.66	-2.36	0.34	0.138
Total chol. (mmol/L)	-0.22 ± 0.12	-0.47	0.03	0.085
LDL-C (mmol/L)	-0.18 ± 0.08	-0.34	-0.01	0.038
HDL-C (mmol/L)	-0.04 ± 0.03	-0.10	0.02	0.220
TAG (mmol/L)	-0.01 ± 0.05	-0.12	0.09	0.787
Glucose (mmol/L)	-0.26 ± 0.12	-0.51	-0.01	0.042
Insulin (µU/mL)	-0.75 ± 1.07	-3.00	1.50	0.493
Systolic BP (mmHg)	1.02 ± 1.56	-2.22	4.26	0.519
Diastolic BP (mmHg)	-1.03 ± 1.15	-3.42	1.37	0.383

* The median change in protein intake (2.0 E%) was used as stratification variable to create two subgroups (subgroup 1 ≤ median change vs. subgroup 2 > median change). Abbreviations: WMD, weighted mean differences.

Table S8. Effect of study design on cardiovascular disease (CVD) risk markers *.

Risk marker	Difference WMD ± SEM	Lower 95% CI	Upper 95% CI	*p*-value
Weight (kg)	1.57 ± 0.65	0.25	2.89	0.021
Total chol. (mmol/L)	0.14 ± 0.13	-0.12	0.41	0.278
LDL-C (mmol/L)	0.09 ± 0.09	-0.10	0.28	0.351

HDL-C (mmol/L)	0.00 ± 0.03	-0.07	0.08	0.895
TAG (mmol/L)	0.08 ± 0.05	-0.02	0.19	0.114
Glucose (mmol/L)	0.18 ± 0.13	-0.08	0.44	0.166
Insulin (µU/mL)	0.58 ± 1.05	-1.61	2.78	0.584
Systolic BP (mmHg)	4.39 ± 2.24	-0.25	9.04	0.063
Diastolic BP (mmHg)	1.81 ± 1.63	-1.57	5.19	0.278

* Changes in cross-over-designed studies were compared to those in parallel-designed studies as reference. Abbreviations: WMD, weighted mean differences.

Table S9. Effect of study duration on cardiovascular disease (CVD) risk markers.

Risk marker	Categories*	WMD ± SEM	Lower 95% CI	Upper 95% CI	p-value
Weight (kg)	>1 month	-1.37 ± 0.75	-2.91	0.17	0.078
	>6 months	-0.92 ± 0.79	-2.53	0.69	0.253
Total chol. (mmol/L)	>1 month	-0.21 ± 0.16	-0.54	0.11	0.189
	>6 months	-0.14 ± 0.17	-0.49	0.21	0.415
LDL-C (mmol/L)	>1 month	0.23 ± 0.12	-0.48	0.01	0.058
	>6 months	-0.15 ± 0.12	-0.40	0.10	0.228
HDL-C (mmol/L)	>1 month	-0.06 ± 0.04	-0.15	0.02	0.113
	>6 months	-0.01 ± 0.04	-0.10	0.70	0.113
TAG (mmol/L)	>1 month	-0.14 ± 0.07	-0.28	0.01	0.058
	>6 months	-0.11 ± 0.07	-0.26	0.04	0.145
Glucose (mmol/L)	>1 month	-0.40 ± 0.29	-1.01	0.21	0.185
	>6 months	0.12 ± 0.30	-0.51	0.74	0.701
Insulin (µU/mL)	>1 month	-2.63 ± 1.76	-6.32	1.07	0.153
	>6 months	0.44 ± 1.79	-3.33	4.21	0.810
Syst. BP (mmHg)	>1 month	-5.17 ± 2.52	-10.4	0.07	0.053
	>6 months	-3.31 ± 2.39	-8.28	1.65	0.179
Diast. BP (mmHg)	>1 month	-2.28 ± 1.85	-6.14	1.58	0.233
	>6 months	-0.88 ± 1.75	-4.52	2.77	0.623

* Study durations of >1 month and >6 months were compared to study durations of ≤ 1 month as reference category. Abbreviations: WMD, weighted mean differences.

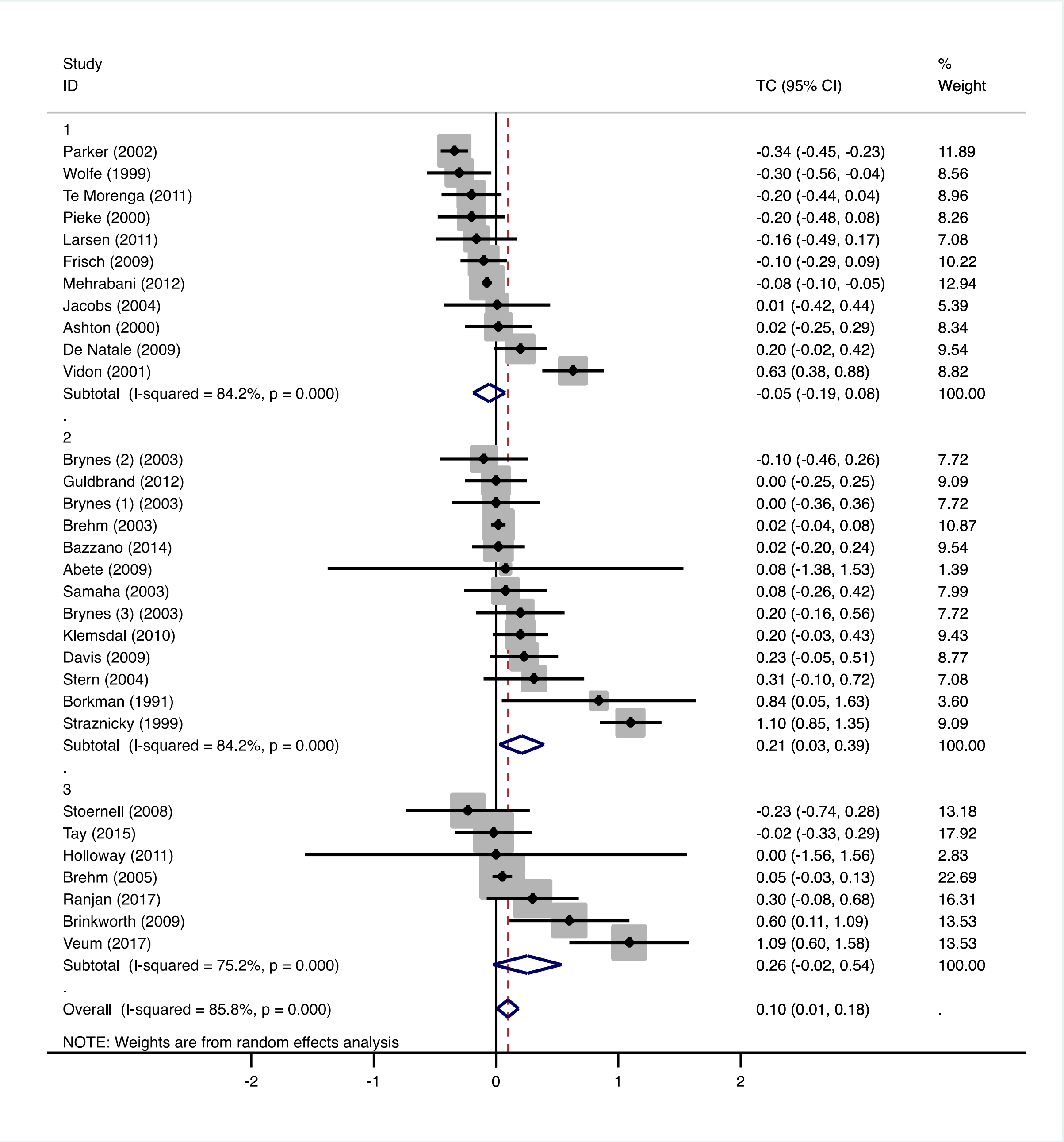

Figure S1A. Forest plots of randomized controlled trials that examined the effects of carbohydrate (CHO) restriction on total cholesterol concentrations. Studies were categorized in group 1 (moderate-low CHO, 40–45 E%), group 2 (low CHO, 40–30 E%), and group 3 (very-low CHO, 30–3 E%). Solid squares represent the weight of individual studies and diamonds represent the weighted mean difference (WMD) in total cholesterol. Effects were calculated using random-effect meta-analysis. No significant differences in total cholesterol were detected between the low-carbohydrate diet (LCD) groups.

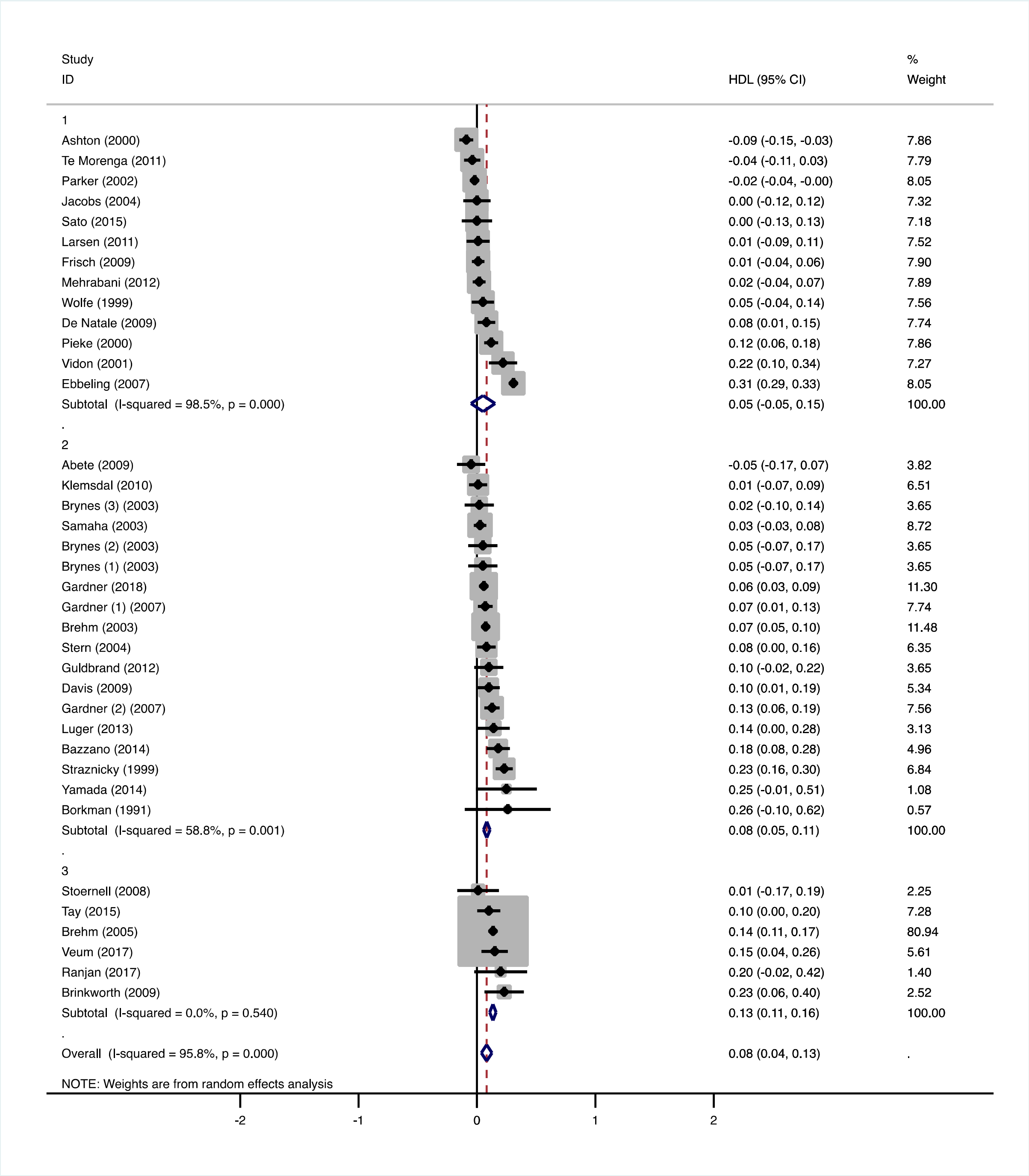

Figure S1B. Forest plots of randomized controlled trials that examined the effects of carbohydrate (CHO) restriction on HDL-C concentrations. Studies were categorized in group 1 (moderate-low CHO, 40–45 E%), group 2 (low CHO, 40–30 E%), and group 3 (very-low CHO, 30–3 E%). Solid squares represent the weight of individual studies and diamonds represent the weighted mean difference (WMD) in HDL-C. Effects were calculated using random-effect meta-analysis. No significant differences in HDL-C were detected between the low-carbohydrate diet (LCD) groups.

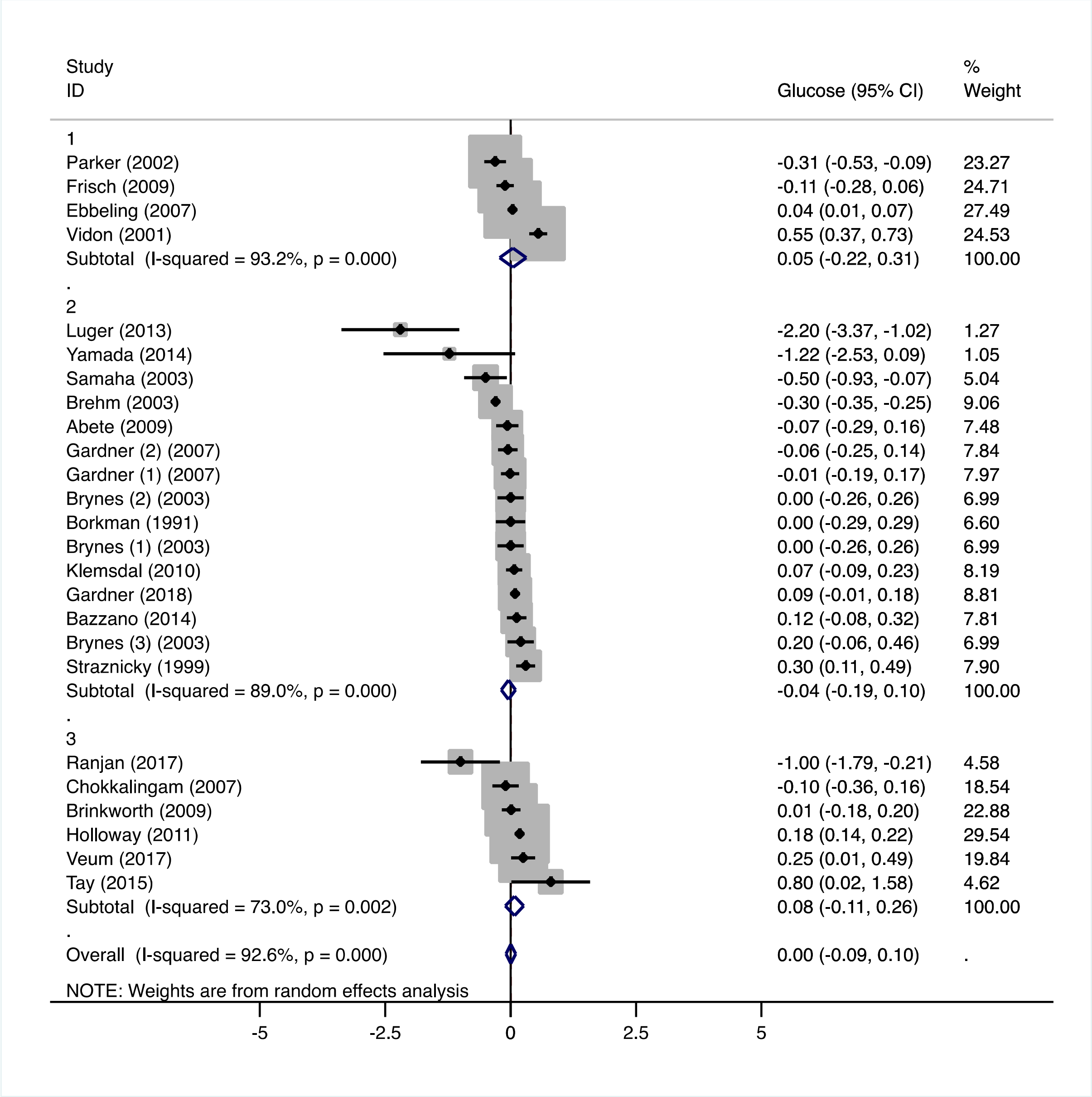

Figure S1C. Forest plots of randomized controlled trials that examined the effects of carbohydrate (CHO) restriction on plasma glucose concentrations. Studies were categorized in group 1 (moderate-low CHO, 40–45 E%), group 2 (low CHO, 40–30 E%), and group 3 (very-low CHO, 30–3 E%). Solid squares represent the weight of individual studies and diamonds represent the weighted mean difference (WMD) in glucose. Effects were calculated using random-effect meta-analysis. No significant differences in glucose were detected between the low-carbohydrate diet (LCD) groups.

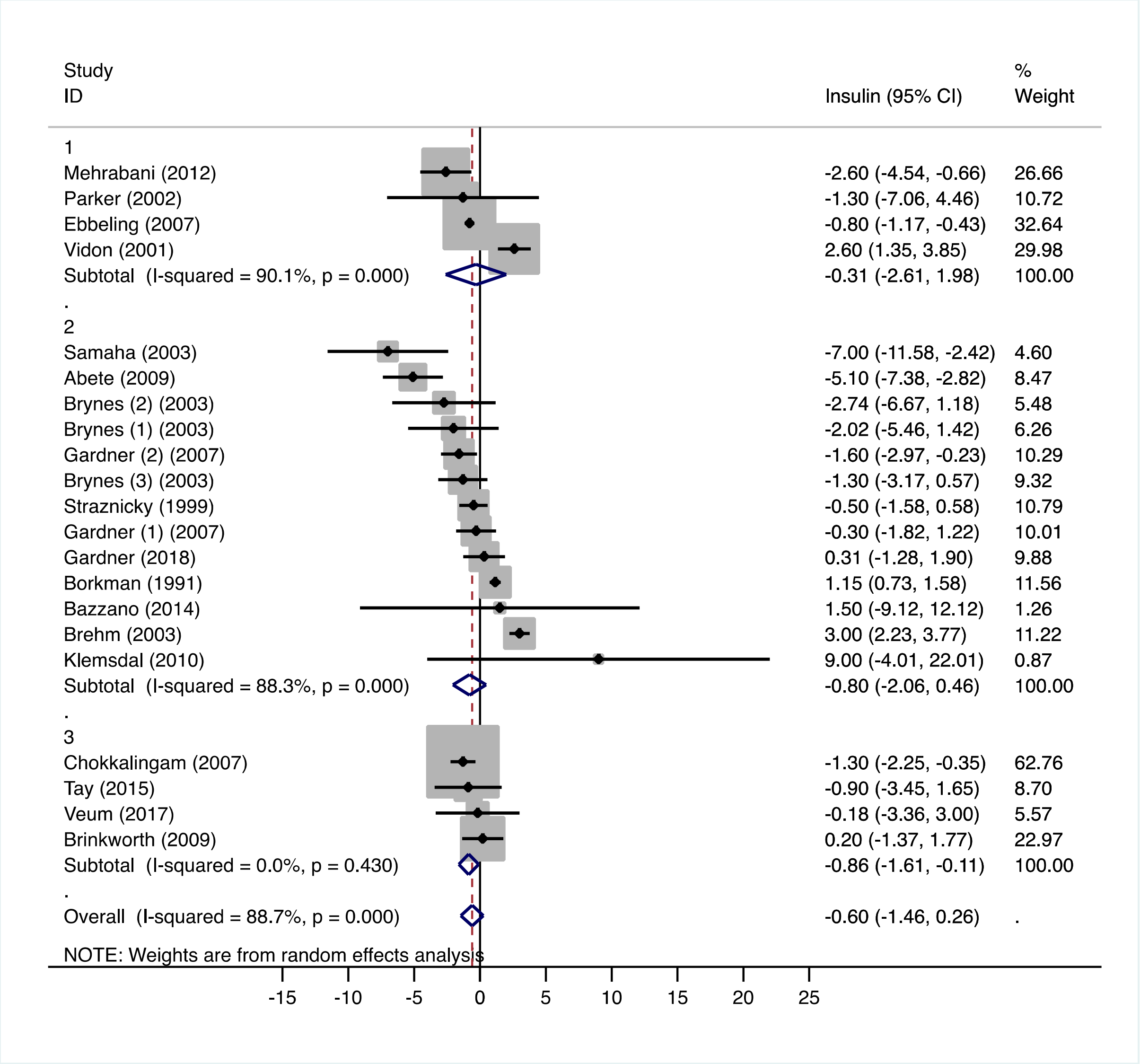

Figure S1D. Forest plots of randomized controlled trials that examined the effects of carbohydrate (CHO) restriction on serum insulin concentrations. Studies were categorized in group 1 (moderate-low CHO, 40–45 E%), group 2 (low CHO, 40–30 E%), and group 3 (very-low CHO, 30–3 E%). Solid squares represent the weight of individual studies and diamonds represent the weighted mean difference (WMD) in insulin concentrations. Effects were calculated using random-(effect meta-analysis. No significant differences in insulin were detected between the low-carbohydrate diet (LCD) groups.

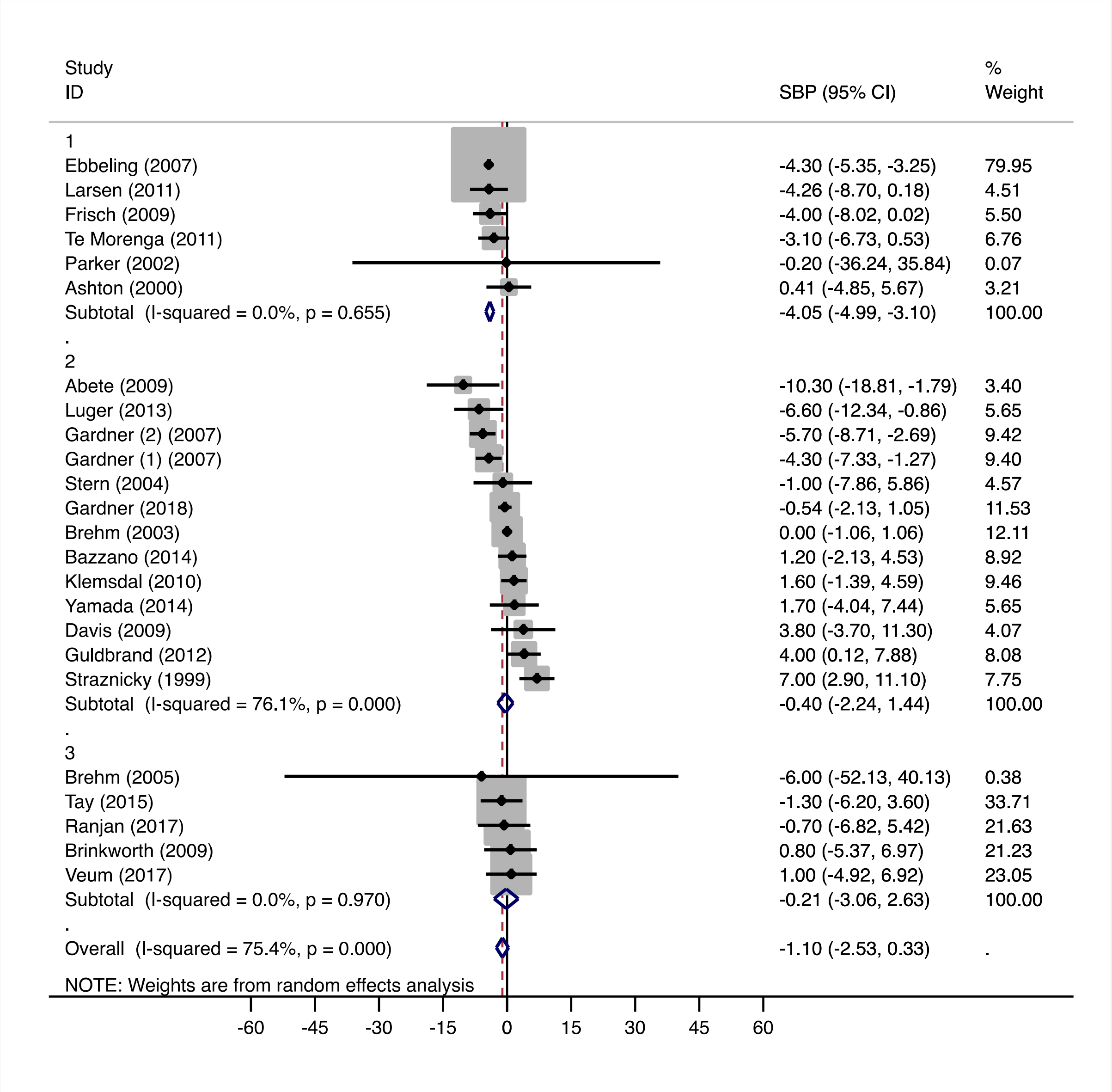

Figure S1E. Forest plots of randomized controlled trials that examined the effects of carbohydrate (CHO) restriction on systolic blood pressure. Studies were categorized in group 1 (moderate-low CHO, 40–45 E%), group 2 (low CHO, 40–30 E%), and group 3 (very-low CHO, 30–3 E%). Solid squares represent the weight of individual studies and diamonds represent the weighted mean difference (WMD) in systolic BP. Effects were calculated using random-effect meta-analysis. No significant differences in systolic BP were detected between the low-carbohydrate diet (LCD) groups.

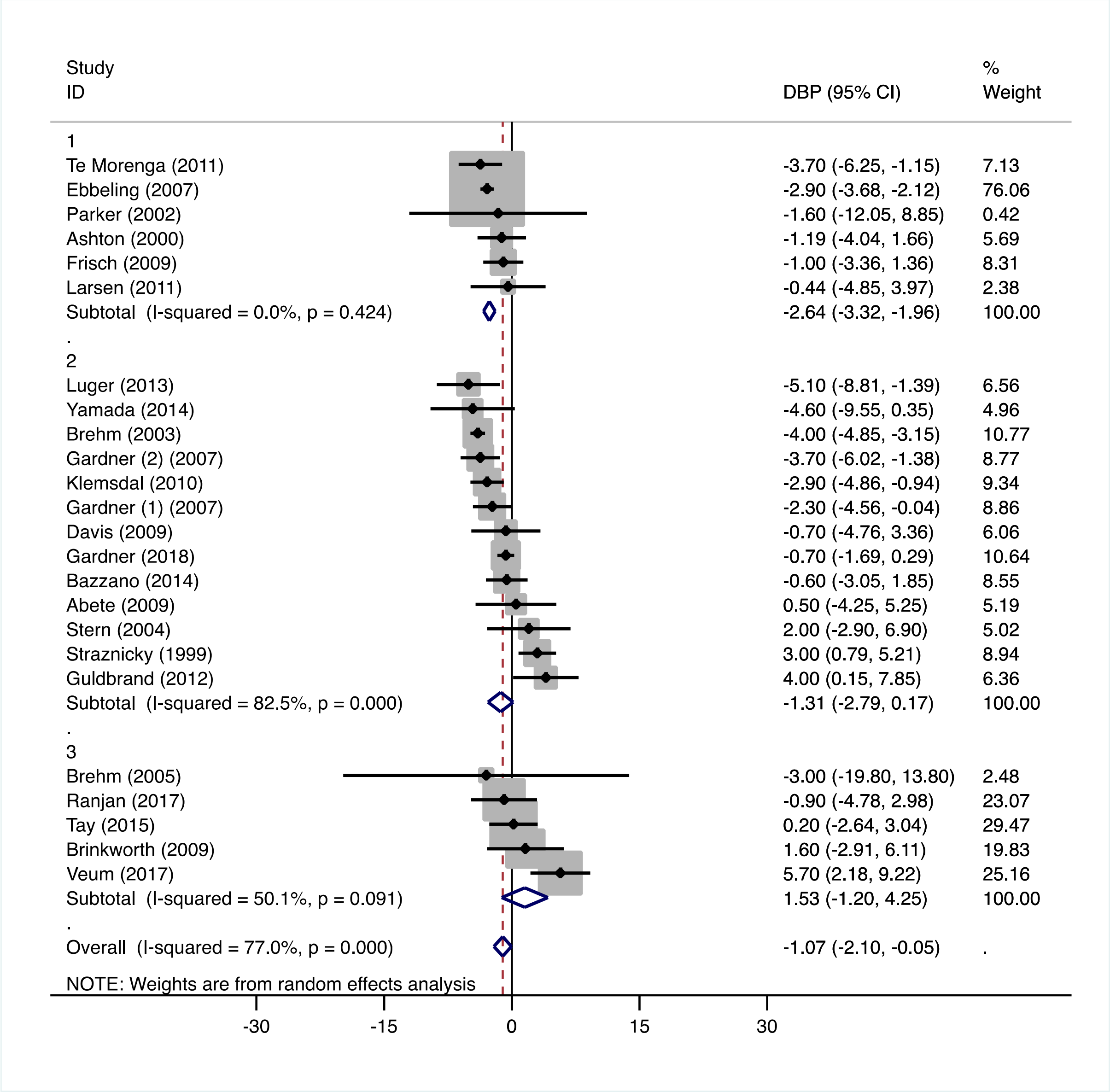

Figure S1F. Forest plots of randomized controlled trials that examined the effects of carbohydrate (CHO) restriction on diastolic blood pressure. Studies were categorized in group 1 (moderate-low CHO, 40–45 E%), group 2 (low CHO, 40–30 E%), and group 3 (very-low CHO, 30–3 E%). Solid squares represent the weight of individual studies and diamonds represent the weighted mean difference (WMD) in diastolic BP. Effects were calculated using random-effect meta-analysis. No significant differences in diastolic BP were detected between the low-carbohydrate diet (LCD) groups.

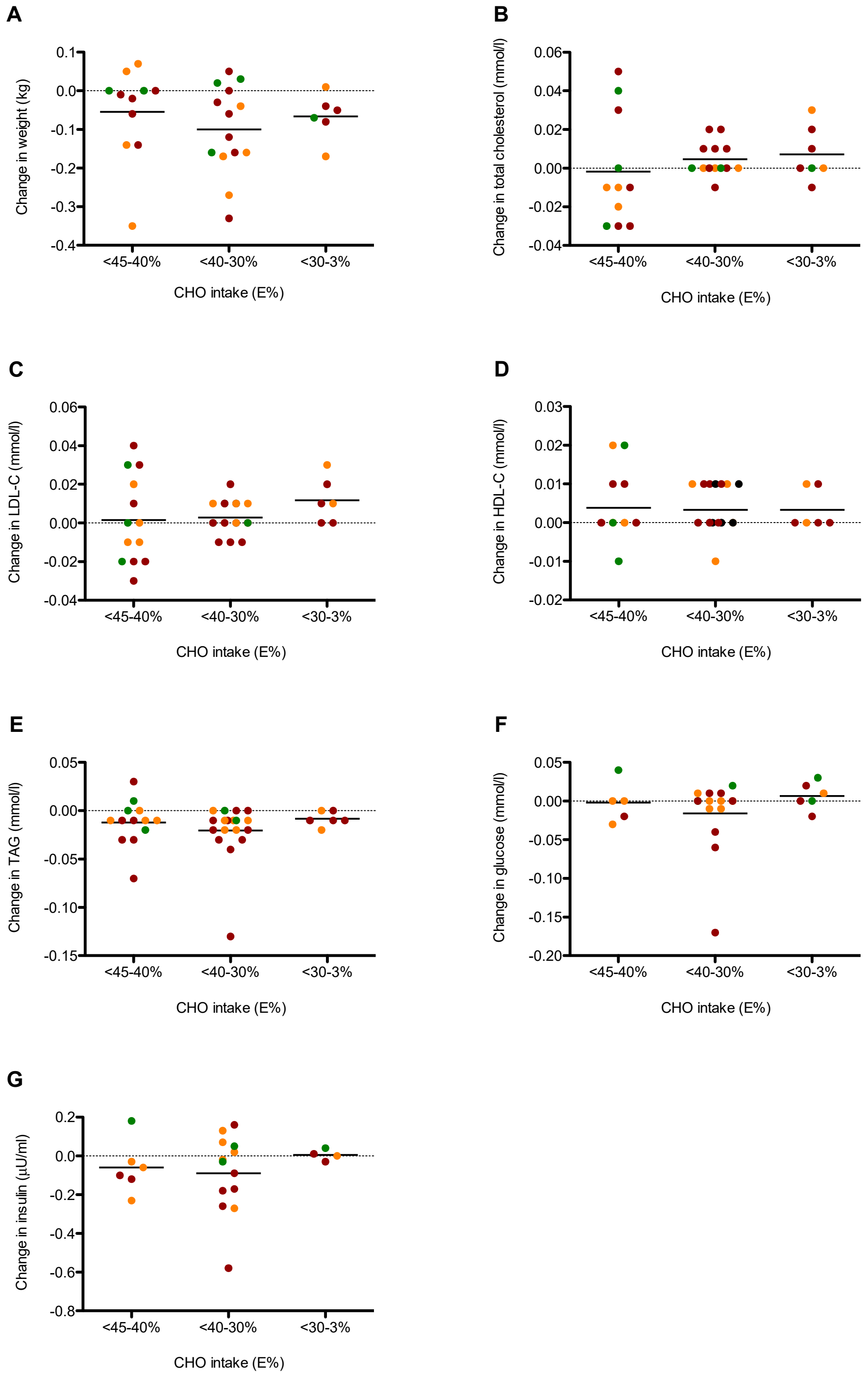

Figure S2. Comparison of the diet effects of the moderate-low (<45–40 E%), low (<40–30 E%), and very low (<30–3 E%) carbohydrate (CHO) groups on (**A**) weight, (**B**) total cholesterol, (**C**) LDL-cholesterol, (**D**) HDL-cholesterol, (**E**) triacylglycerol, (**F**) glucose, and (**G**) insulin. Data is expressed as mean changes per percentage reduction in carbohydrates. Significant results are represented by squares. The health status of the study population is indicated in green (healthy), orange (overweight/obese), and red (metabolically impaired, including type 2 diabetes and metabolic syndrome).

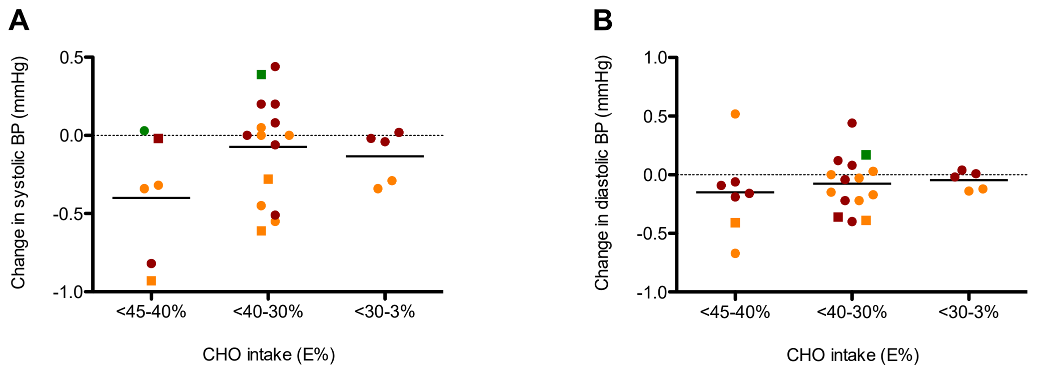

Figure S3. Comparison of the diet effects of the moderate low (<45–40 E%), low (<40–30 E%), and very low (<30–3 E%) carbohydrate (CHO) groups on (**A**) systolic and (**B**) diastolic blood pressure. Data is expressed as mean changes per percentage reduction in carbohydrates. Significant results are represented by squares. The health status of the study population is indicated in green (healthy), orange (overweight/obese), and red (metabolically impaired including type 2 diabetes and metabolic syndrome).
